# Supplementary figures and images for: Rhododendron chrysanthum’s Primary Metabolites Are Converted to Phenolics More Quickly When Exposed to UV-B Radiation
Source: Biomolecules. 2023 Nov 24;13(12):1700. doi: 10.3390/biom13121700 (PMC10742171; doi:10.3390/biom13121700)

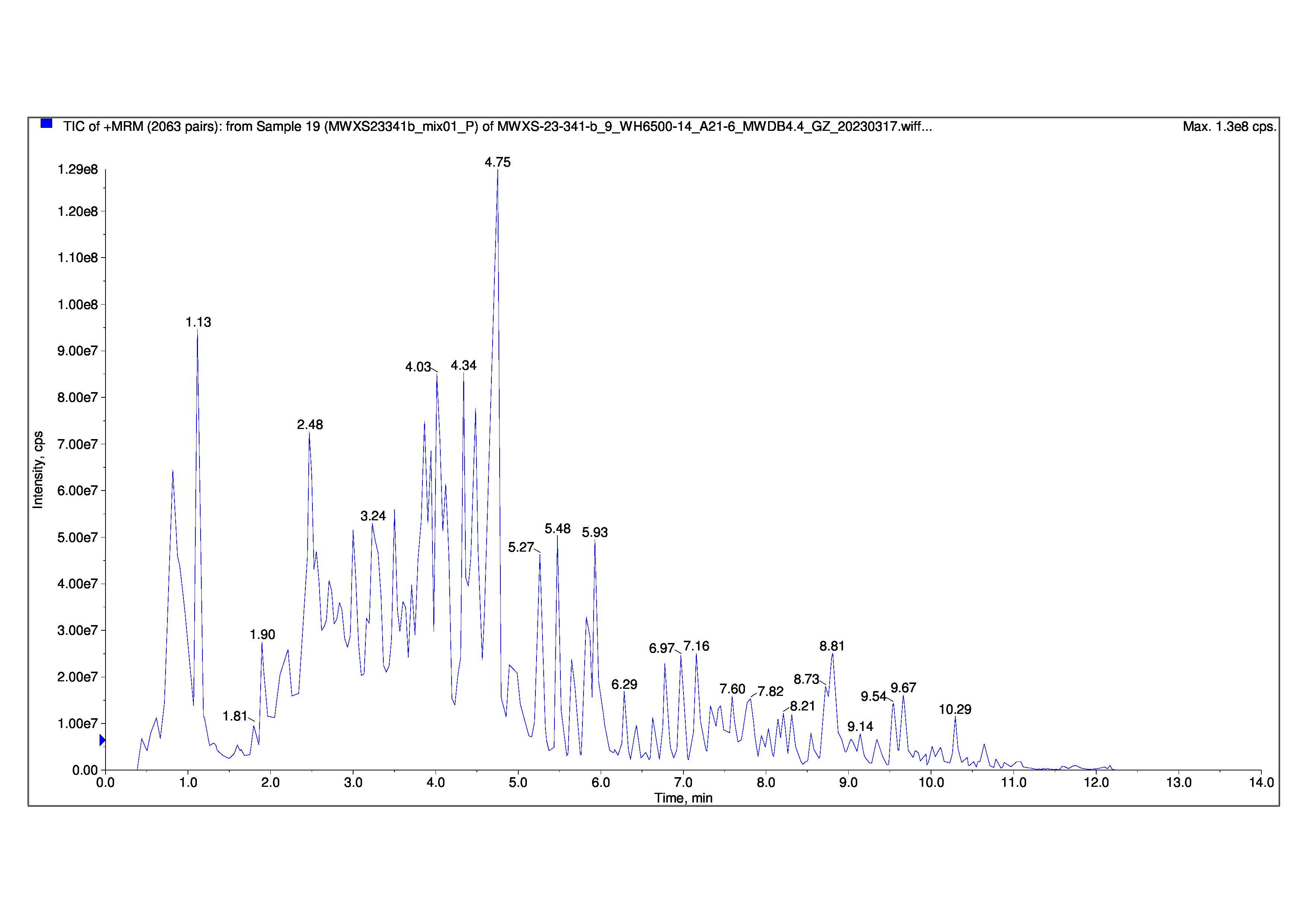

Supplement: Supplementary file 1 [file biomolecules-13-01700-s001.zip › Figure S1.Total ion flow diagram in positive ion mode.png]

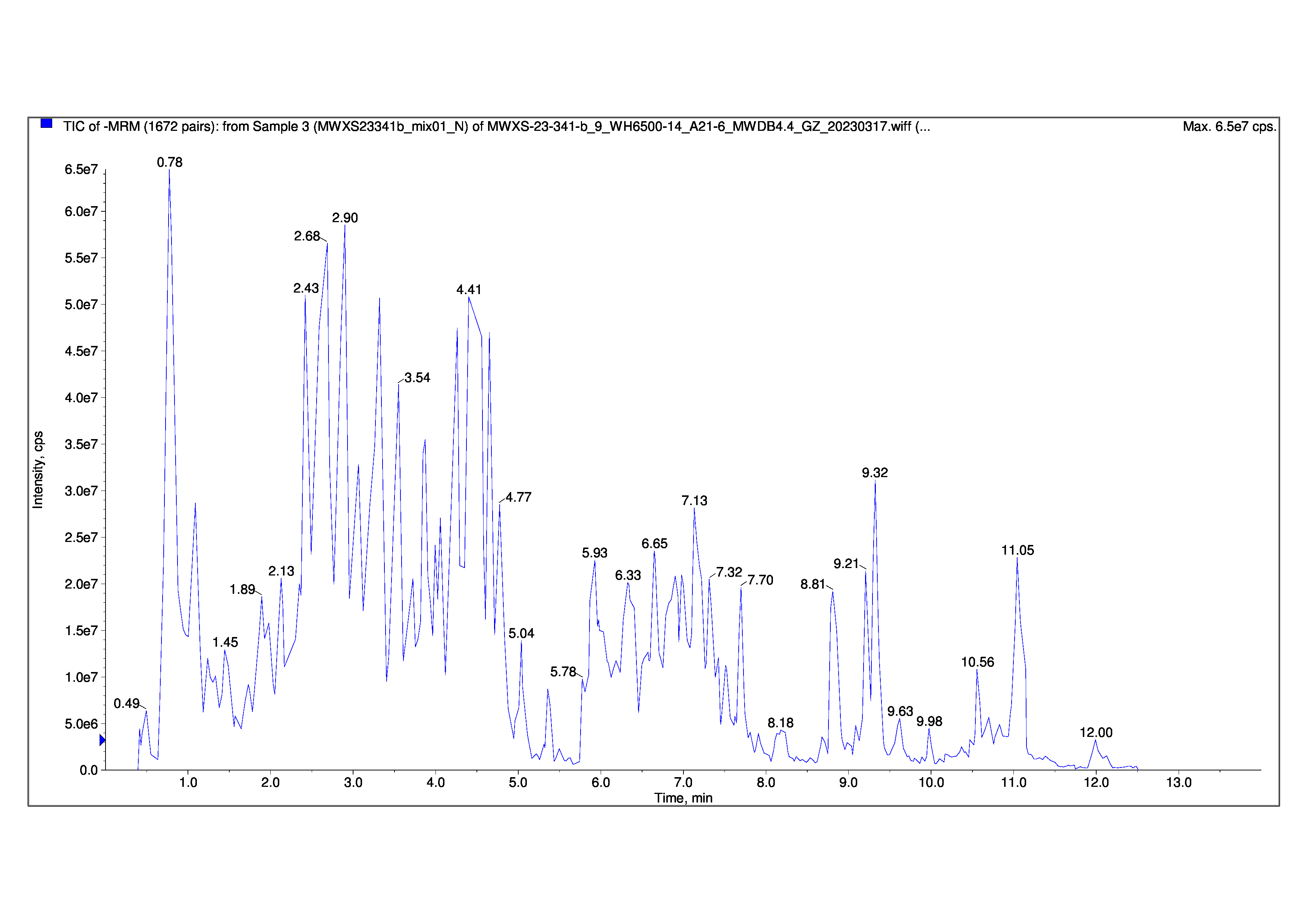

Supplement: Supplementary file 1 [file biomolecules-13-01700-s001.zip › Figure S2.Total Ion flow map in negative Ion mode.png]
